# In-hospital care prior to assisted and unassisted suicide in swiss older people: a state-level retrospective study

**DOI:** 10.1186/s12877-019-1325-6

**Published:** 2019-11-06

**Authors:** Nathalie Dieudonné-Rahm, Sandra Burkhardt, Sophie Pautex

**Affiliations:** 1 Department of Rehabilitation and Geriatrics, Division of Palliative Medicine, Geneva University Hospitals, Chemin de la Savonnière 11, 1245 Collonge Bellerive, Geneva, Switzerland; 2 Division of Legal Medicine, Geneva University Hospitals, Geneva, Switzerland

## Background

Suicide is a major public health issue, affecting all ages and is expected to grow as life expectancy increases [1, 2]. The incidence of suicide is particularly high among older males aged 65 and older [3]. The rate of suicide in the oldest group (ages 85+) is over four times higher than overall rate of suicide in Switzerland or in the US; moreover, this age group shows an increase in suicide rates in almost all countries [3, 4].

Elderly persons have a higher risk of completed suicide compared with younger age groups. Their frailer health status should not be considered as the main contributing factor to this excess of mortality. Elderly use more deadly methods and are less likely to be found and rescued because of the scarcity of warning signs when planning and carrying out the lethal act [5].

Whereas for the majority of the countries in the world, assisted suicide is not an option, in Switzerland, this process is permitted. In this country, assisted suicide counts for about 1% of deaths, with people 65 years and older having the highest rates of assisted suicide [4, 6, 7]. A person can be granted access to assisted suicide when the following two conditions are met: to have decisional capacity and to be able to perform the fatal act oneself. In addition, other persons implicated in this process should have no selfish motive. The Swiss legal framework permits right-to-die societies to assess and decide on the acceptability of a request, without restrictions regarding the source of suffering.

The Swiss Academy of Medical Sciences ethical guideline for end-of-life care issued in 2013 required that the underlying disease implies that the end-of-life is expected to be near. However, since 2014, right-to-die organizations in Switzerland have considered requests for assisted suicide, in the case of unbearable suffering or age-related changes, even in the absence of terminal illness [8, 9].

Since assisted suicide became relatively easier to access in Switzerland, we anticipated that, over the years, an increasing number of people with chronic illnesses would carry out assisted suicide to end their suffering. We additionally hypothesized that there would be a decrease in unassisted suicides with the widening of indications by right-to-die organizations in 2014.

Suicide in late life is known to be complex and multifactorial. Some factors have been found to influence both types of suicides in the elderly, such as constricted social networks (loneliness, social exclusion, bereavement), mood disorders and cognitive impairment [10–13]. It has been found in recent systematic reviews that physical illness and functional impairment are important suicide risk factors in the elderly. Furthermore personality disorders that lead to an inability to adapt to the changes occurring in late life are more likely to be found in older adults who attempt or complete suicide than in their younger counterparts [14–18]. These findings suggest that the elderly who contemplate suicide would be particularly sensitive to any worsening of their quality of life. Older age suicide may have rational grounds, following a process of deliberation and holistic self-assessment of irremediable impairment of current or future ones-quality of life [19]. Indeed, elderly persons may contemplate suicide due to being tired of life or considering that their life is complete. Neoliberalism, technology, and changing attitudes related to the legalization of assisted death in some countries are sociological trends thought to influence rational suicidal thoughts [20].

However, elderly prone to unassisted and assisted suicide differ in many ways.

Unassisted suicide in the elderly often follows impulsive decision-making during major depressive or mixed illness episodes [21]. Unassisted suicide is known to be strongly associated with previous suicide attempts, mood disorders (e.g. depression, bipolar disorder), vulnerability in the context of borderline or other personality disorders, presence of impulsive-aggressive traits, hopelessness and substance use [22–26]. Individuals with mental and behavioral disorders generally report having a poor perceived quality of life, as they are at greater risk of experiencing marginalization, poverty and stigma, and show a higher incidence of suicide than the general population [21, 22, 27, 28].

On the other hand, the typical assisted suicide decedent profile has been described as a single or divorced woman with tertiary education, without religious affiliation, affected by cancer or any other chronic illness [29]. Persons who choose to opt for assisted suicide are supposed to make an informed choice about how and when to die. They are evaluated for their decisional capacity beforehand and are imposed a time for reflection before making their final decision. Thus, the decision to end one’s life via assisted suicide may become an option, especially when facing or fearing chronic illnesses’ consequences. Indeed, chronic illnesses can leave patients in a debilitative state, that no longer enables the necessary self-determination to end their own lives. Fears of indignity, of being a burden, of losing autonomy or losing control as well as tiredness of living have also been reported as reasons for requesting assisted suicide [12, 13].

We thus anticipated that assisted suicide would be associated with more frequent somatic health problems leading to intense in-hospital care, whereas unassisted suicide would more frequently occur in cases with mental disorders. This divergence would explain older suicide victims’ different utilization of health care. Increased health care intensity would increase in assisted suicide but not unassisted suicide, since it adds to individuals’ experienced burden. Moreover, higher utilization of health care facilities could increase the opportunity for suicide prevention by screening and treating more elderly patients with severe mental illness who would carry out impulsive suicidal acts.

Late-life suicide remains understudied although the characterization of individuals would be central to reduce the progression of suicidal thoughts to acts and to improve elderly people’s quality of life. Assisted suicide research has focused on sociodemographic characteristics and general diagnoses, mostly disregarding individuals’ previous experience with healthcare. Examining health care experience seems nevertheless important as fears of medical treatments, hospitalizations and nursing home stays based on prior experience appear to be dramatic triggers of the contemplation phase of assisted suicide [30].

There is strong evidence for an increasingly high and aggressive intensity of care during the last 12 months of life [31–38]. High intensity of care can be experienced as impacting quality of life negatively, as being futile and inconsistent with the patient’s wishes [39]. Hospital care is also associated with an increased risk of depression in older patients [40–42]. Medicalization at the end of life may ultimately alter the patient’s desire to live and influence their wish or decision to die [43]. Furthermore, high levels of care may cause older adults to fear their lives would be prolongated against their will [39, 43].

Based on these results, the authors have made the hypothesis that, the level of care may be experienced by older patients as a burden or as repeated negative events altering their quality of life and thus may be an important influencing factor for carrying out assisted suicide.

Prior hospital care intensity may be a positive predictor of assisted suicide relatively to unassisted suicide. If confirmed, this association would further increase with age, somatic diseases and hospitalizations.

We tested the hypotheses in a state level retrospective study conducted in Geneva, Switzerland. The study employed data from individually linked hospital medical and forensic records to assess the strength of the associations between high intensity of care (for mental health or somatic reasons) received by patients aged 65 years or older prior to their assisted suicide or unassisted suicidal act and to determine whether these associations are modified by patients’ characteristics and diseases or by the type of hospital care experienced.

## Methods

### Design and data sources

This retrospective study included all consecutive persons aged 65 years or older who opted for assisted or unassisted suicide and were examined after their death at the University of Geneva Centre of Legal Medicine between January 2010 and December 2016.

Assisted and unassisted suicide cases were identified from death certificates and confirmed by the Geneva police and the unit of forensic medicine.

Two different data sources were used: 1) results of forensic autopsies, mandatory medical certificates attesting the patients’ known disease(s) and patients’ motivation letters addressed to the assisting right-to-die association; as well as 2) computerized medical charts stored at the Geneva University Hospitals.

#### Ethics and consent to participate

The study was approved by the Geneva branch of the Swiss Ethics Committee in August 2016, with protocol number 2016–00966. No informed consent from patient relatives was required by the ethics committee as this was a retrospective study of health and medico-legal data routinely collected after assisted suicides.

### Patient characteristics’ and hospitalization related data’s collection

For deaths classified as suicide, the following factors were registered from forensic data: age, gender, type and year of suicide. Using patients’ medical records, we further collected data related to marital status (single, married, divorced or widowed), place of living before death (home or institution), main diseases and comorbidities. Diagnoses were coded using ICD-10 (international classification of diseases, 10th revision). Diagnoses were divided into 15 broad categories: infections; neoplasms; diseases of the blood and immune system; endocrine, nutritional and metabolic diseases; mental and behavioural disorders; nervous system diseases; eye and adnexa diseases; ear and mastoid process diseases; circulatory system diseases; respiratory system diseases; digestive system diseases; skin and subcutaneous tissue diseases; genitourinary system diseases; congenital malformations and chromosomal abnormalities; diseases not elsewhere classified.

Acknowledging previous definition criteria of high intensity of care at end-of-life [44, 45], the following health care measures recorded during the last year of life were collected to assess intensity of care. Hospitalizations (in general wards or in psychiatry), emergency department visits, intensive care unit admissions, surgical interventions were also recorded.

### Statistical analysis

Patient characteristics were described using mean values and standard deviations for quantitative variables and frequencies and percentages for categorical variables. Wilcoxon signed-rank test was used for age comparison between both groups.

Differences in other sociodemographic variables, mode of suicide (assisted or unassisted) and types of received care between the two groups of patients were assessed using proportions and compared using the chi-square and Fisher’s exact test, when the expected numbers are smaller than five. Reported *p*-values are two-sided.

All factors related to elderly suicide were prespecified according to our hypotheses. All factors were tested in univariate analysis then selected in the multivariate model when *P*-value was below .20. A multivariate logistic regression was then performed to investigate the association between assisted suicide and intense hospital care variables (hospitalizations, ICU admissions, surgery, emergency department use), adjusting for diagnoses of cancer and/or non-oncologic somatic comorbidities and/or mental and behavioral disorders. We selected variables to obtain a parsimonious model keeping only factors associated with the outcome when the *P*-values were < .05. Statistical analyses were performed using Stata Statistical Software: Release 14 (Stata Corp.*,* College Station, TX, USA).

## Results

**Fig. 1:**
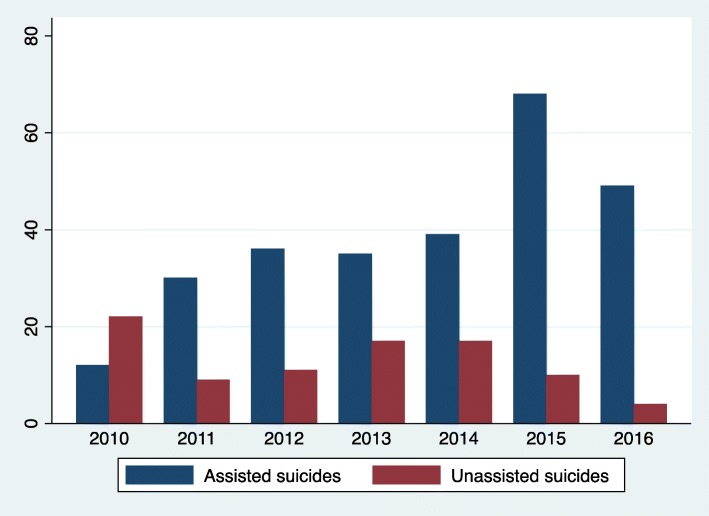
Evolution of elderly suicide deaths numbers registered in Geneva, Switzerland, 2010 to 2016

Figure 1 shows the progression of suicide deaths of older adults in Geneva between 2010 and 2016. During the study period, 359 people died in the group aged 65 and more in Geneva; the majority being assisted suicides (*n* = 269, 75%). Deaths by assisted suicide globally increased by 200%. The proportion of assisted suicide among overall suicides significantly increased in years 2015–2016 compared to 2010–2014 (152 of 228, 66.6%, versus 117 of 131, 89.3%, *P* < .0001); the highest increase of numbers of assisted suicide deaths being observed in 2015.

### Decedents characteristics

All elderly who died by suicide were successfully linked to their electronic medical health records. Results of univariate analyses confirm that individuals who carried out assisted suicide differ from peers who die by unassisted suicide on demographic characteristics and illnesses. Table 1 summarizes demographic characteristics in both groups.

**Table 1:** Demographic characteristics in elderly suicide decedents, Geneva, Switzerland, 2010–2016

Characteristic	All *n* = 359	Unassisted suicide *n* = 90	Assisted suicide *n* = 269	*P*-Value
Age: Mean (SD)	80 (8)	76 (7)	81 (8)	<.001
Male sex-no.(%)	156 (43.4)	57 (63.3)	99 (36.8)	<.001
Marital status-no.(%)				.136
Married	132 (36.7)	33 (36.6)	99 (36.7)	
Widowed	97 (27)	18 (20)	79 (29.3)	
Divorced	62 (17.2)	18 (20)	47 (17.4)	
Single	68 (18.9)	21 (23.3)	42 (15.6)	
Place of living-no.(%)				.023
Home	334 (93)	87 (96.6)	247 (91.8)	
Nursing home	24 (6.7)	2 (2.2)	22 (8.2)	
Unknown	1 (0.2)	1 (1.1)	0	

Assisted suicide deaths are associated with female gender (63.2 vs. 36.8%; *p* < .001), older age (mean age 81 vs. 76; *p* < .001) and the fact of being a nursing home resident (8.2 vs. 2.2%; *p* = .023) but, do not seem to be influenced by marital status. (Table 1).

Diagnoses retrieved by cross-referencing our different sources are summarized in Table 2. Half of the sample was known to have diseases of the circulatory system in both groups. Mental and behavioural disorders were present in two-thirds of individuals carrying out unassisted suicide while in less than one-third of assisted suicide cases. Prevalence of cancer and nervous system diseases was significantly higher in the group who died by assisted suicide than in the other group. (Table 2) Diseases of the blood and immune system, of eye and adnexa diseases, ear and mastoid process diseases, skin and subcutaneous tissue diseases as well as infections, congenital malformations and chromosomal abnormalities were found in less than 10% of patients.

**Table 2:** Diagnoses collected in elderly suicide decedents, Geneva, Switzerland, 2010–2016

Diagnose	All *n* = 359	Unassisted suicide *n* = 90	Assisted suicide *n* = 269	*P*-Value
Circulatory system -no. (%)	181 (50.4)	51 (56.6)	142 (52.7)	.120
Mental and behavioural disorders -no. (%)	136 (37.8)	69 (65.5)	77 (28.6)	< .001
Neoplasms -no. (%)	123 (34.2)	13 (14.4)	110 (40.8)	< .001
Nervous system -no. (%)	100 (27.8)	12 (13.3)	88 (32.7)	< .001
Musculoskeletal system and connective tissue -no. (%)	86 (23.9)	14 (15.5)	72 (26.7)	.031
Digestive system -no. (%)	72 (20)	14 (15.5)	58 (21.5)	.218
Respiratory system -no. (%)	52 (14.4)	14 (14.13)	38 (15.5)	.739
Endocrine, nutritional, metabolic -no. (%)	50 (13.9)	17 (12.27)	33 (18.8)	.116
Genitourinary system -no. (%)	45 (12.5)	10 (11.1)	35 (13)	.716

### Hospital care

Table 3 summarizes hospital care received during the year prior to death comparing elderly who died by assisted suicide to their peers who died by unassisted suicide. Data collected from hospital charts show that assisted suicide decedents underwent a significantly higher intensity of hospital care during their last year of life compared to those who took their own lives without assistance. Among patients who engaged in assisted suicide, decedents were significantly more likely to receive inpatient care during the year prior to death in general wards, undergo more frequently surgery and use more frequently emergency department, compared to the other group. More than one-third of them visited the emergency department and nearly one of five had had surgical interventions. Only few had been admitted to an intensive care unit.

**Table 3:** In-hospital care received by elderly suicide decedents in the year prior to death, Geneva, Switzerland, 2010–2016

Type of care	All *n* = 359	Not-assisted suicide *n* = 90	Assisted suicide *n* = 269	*P*-Value
Hospitalizations - no. (%)	186 (51.8)	30 (33.3)	156 (57.9)	<.001
Hospitalizations in general wards- no. (%)	172 (47.9)	17 (18.8)	155 (57.6)	<.001
Hospitalizations in psychiatry- no. (%)	24 (6.6)	19 (21.1)	5 (1.8)	<.001
Ambulatory emergency department use - no. (%)	110 (30.6)	11 (12.2)	99 (36.8)	<.001
Surgery - no. (%)	66 (18.3)	3 (3.3)	63 (23.4)	<.001
Intensive care unit admissions - no. (%)	15 (4.2)	3 (3.3)	12 (4.5)	.770

Table 4 shows the associations between assisted suicide, age, gender, diagnoses and different markers of intense hospital care. Older age, female sex, cancer and somatic diseases were associated significantly and independently with assisted suicide, whereas the presence of mental illness was associated negatively and independently with assisted suicide. The results of logistic regressions performed indicated that hospitalizations with surgery (OR = 20; *p* < .001) and without surgery (OR = 3; *p* = .006), during the year prior to death, were factors more frequently associated with assisted suicide, independently of the presence of mental and behavioral disorders, cancer or other somatic diseases, than with unassisted suicide.

**Table 4:** Results of logistic regressions examining the associations between elderly assisted suicide and hospital care in the year prior to death, adjusting for age, sex and diseases

Variable	*Odds ratio*	95%CI	*P-Value*
Age	1.08	1.04–1.13	.001
Female sex	5.64	2.85–11.16	<.001
Hospital care (reference: no hospital care)			.001
Ambulatory emergency department visit	1.11	0.26–4.64	.886
Hospitalisation without surgery or intensive care unit stay	3.18	1.45–6.98	.006
Surgery	20.17	3.86–105.34	<.001
Intensive care unit stay	2.44	0.57–10.47	.228
Neoplasm(s)	6.88	3.05–15.53	<.001
Somatic disease(s) excepted neoplasms	3.65	1.78–7.49	<.001
Mental or behavioural disorder	.17	.08–.36	<.001

## Discussion

As hypothesized, our study shows a substantial progression in assisted suicide deaths between 2010 and 2016, for both men and women 65 years and older, in Geneva, with a peak in 2015. This peak most likely reflects the expansion of eligibility criteria for requesting assisted suicide to non-terminal diseases decided by right-to-die organizations in 2014. The count of assisted suicides in retirement homes reached a peak the same year, thus participating to this phenomenon. The social impact of the increase in assisted suicide on families should not be disregarded as some studies has shown a higher prevalence of post-traumatic stress disorders and depression among family members [46].

Elderly assisted suicide decedents were more likely to receive highly intensive care than peers who died by unassisted suicide, with these positive associations being stronger among the oldest women suffering from somatic illnesses living at home or in a nursing home as previously reported [47]. First, the oldest elderly may be more tired of living than their younger counterparts, and thus more at risk of rational suicide. The oldest old living at home may suffer from isolation and social exclusion, that are well known risk factors for assisted suicide [4]. In the other hand, the fact that we found more assisted suicide than unassisted suicide in nursing homes, could be explained by a narrower contact with caregivers as well as the generally worse level of functioning of residents that prevent carrying out a suicidal act on their own.

Mental and behavioural disorders as well as poor use of hospital care were more likely to be found in the unassisted suicide group compared to the assisted suicide group. This result is consistent with the fact that previous reports showed that patients with mental disorders are at risk to receive poorer access and quality care as a result of mental illness-related stigma [28, 48]. Furthermore, help-seeking behaviour is affected among patients with mental illness [48]. Individuals with mental and behavioral disorders often lack the necessary level of planning and organization to engage in assisted suicide procedures when they consider ending their life, and therefore would engage in more impulsive suicide attempts even when an assisted way of dying is available [21].

As mental health disorders are rarely found in assisted suicide decedents records, screening and treating mental illness (e.g. depressive state) would not be sufficient to reduce assisted suicide death. Further research should therefore examine whether it is of interest to look for the determinants of poor quality of life in this group to enhance their autonomy.

Somatic diseases such as cancer and non-malignant chronic diseases were more frequently associated with assisted suicide than unassisted suicide as previously reported [49]. Diseases of the circulatory system were the most prevalent diagnoses present in both groups whereas cancer was the second main diagnosis found in assisted suicide decedents. Cardiovascular diseases such as coronary heart disease, hypertension and stroke are strongly linked with depression and loneliness, which could be linked with both types of suicide [50].

Hospitalization rates of people aged 70 and more were at 20% in Geneva state according to 2016 statistics, which is comparable to the values found in our study for the unassisted suicide group (18.8%). In our study, this percentage was by comparison about three times lower than the rate observed among people who died by assisted suicide (57.9%) [51].

The association between assisted suicide and hospitalization could have reflected the physical, psychological, existential, social and/or financial burden associated with severe somatic diseases. Patients with advanced cancer as well as those facing non-oncologic diseases are indeed at high risk of receiving intensive levels of care at the end of life [52].

However, the discrepancy in hospital care utilization between people who died by assisted versus unassisted suicide did not appear to be fully accounted for by the difference observed in the diagnoses nor the other tested characteristics. As hospitalization and surgery are independently associated with assisted suicide, our findings augment existing evidence that these markers of high level of care impact on the wish to live negatively.

This phenomenon could be influenced by changes in the quality of life during hospitalization, particularly after surgery or other contacts with health care professionals.

It has been indeed demonstrated that hospitalizations put elderly patients at risk of disability, depression and cognitive loss. Moreover, delirium which is very common especially after surgery, may be persistent after hospitalizations [53–57].

No evidence was found to support associations between the type of suicide and emergency department unit admissions without hospitalizations or ICU admissions. One explanation might be that emergency department ambulatory care is due to unstable situations where patients find support and receive reassuring punctual care that does not have a lasting negative impact on their quality of life. In addition, ICU admissions, which are proven to impact late-life quality of life negatively, were events that occurred too rarely in our study to confirm their potential negative impact on quality of life and the desire to live meaningfully [42].

### Strengths and limitations

To our knowledge this is the first study to show how surgery and hospitalization during the year prior to death are strongly associated to assisted suicide in older patients. The study’s novelty resides in the quantitative comparison of assisted and unassisted suicide older decedents in a large state level sample.

The increase observed in assisted suicide deaths in elderly in Geneva is in line with the progression of the number of cases of assisted suicide deaths described in other countries of Europe and in American states with long-time acceptance and experience in assisted suicide. Furthermore, no evidence was found to support an association between the progression of assisted suicide in Switzerland and any shift in potential socio-economic factors [6].

Since this study was limited to the Geneva area, it could be argued that our results may not be applied to other contexts, because end-of-life intensity of care is known to vary substantially within economic and sociodemographic conditions, health care supplies and institutional norms [12, 48]. Indeed, the intensity of end-of-life care may be higher in Geneva than in other countries or parts of Switzerland, partly because this area has a higher number of beds per individual (with 5.2 beds/1000 individuals in Geneva in 2016) compared to the rest of Switzerland (4.5 beds/1000 individuals in Switzerland in 2015) [49]. However, it has been shown that the increase in cases of assisted suicide is the highest in the French-speaking region of Switzerland, which includes Geneva. This fact reinforces our hypothesis that a high intensity of care is associated with assisted suicide in elderly.

While other studies focused on diseases reported by patients to request assisted suicide or recorded on death certificates, this study provides a description of main diagnoses found in patients electronic medical records and forensic investigations. Diagnoses and measures of care were found in 100% of individuals who carried out suicide during the study period due to the availability of forensic records. High quality data was extracted from forensic documents and hospital records and linked at an individual level, which can be considered as more accurate than administrative or insurance data in mirroring the reality and complexity of the care received by patients and reducing the ecological fallacy bias. However, the fact that data related to intensity of care was only collected at our institution constitutes a limitation.

Finally, the key outcome measures of this study are markers of hospital use during the year prior to death: hospitalizations, emergency visits, surgery, ICU admissions. As discussed by Wong and O’Hare, measures of healthcare use and treatment practices are probably inaccurate markers for intensity and quality of care from the patient and family points of view [58]. End-of-life intensity of care delivered in hospitals probably corresponds to only the emergent and measurable part experienced by patients and families. Most likely, we should consider the patient or their family as the most reliable source of information regarding this issue. Other potential risk factors contributing to end-of-life intensity of care should also be explored by questioning elderly patients, who are suffering from chronic diseases.

### Implications

Studies are increasingly needed to determine the extent to which requests for assisted suicide are related to the burden of high level of care and the extent to which health care may cause suffering.

Considering and honouring patients’ goals and wishes regarding end-of-life care appears to be crucial and may help to reduce suicidal thoughts in this population. Advance care planning and earlier referral to a palliative care team should be encouraged to help slow down intensity of end-of-life care and to provide care that better matches patients’ wishes.

Considering hospital-associated risks in older people is crucial to develop strategies to provide optimal care, to promote alternatives to hospitalization and to enable earlier discharge. Caregivers working in hospitals should be better involved in exploring wishes to hasten death, as well as patients’ quality of life as a consistent proportion of older assisted suicide decedents were admitted in hospital within the 12 months prior to suicide. Health care professionals in charge of patients in perioperative setting should be aware that surgery may impact quality of life negatively and wishes to hasten death positively. Initial and continuing education should be strengthened to enable those professionals to develop knowledge and skills in the field of geriatrics and palliative care. Providing assessment by mental health professionals and palliative care specialists as well as ensuring continuity and safe transitions of care could be some key points to improve health care as well as quality of life conditions that could provide older people with alternatives to suicide.

## Conclusions

Compared to individuals who carried out unassisted suicide, older patients who opted for assisted suicide receive significantly more intense hospital care during their last year of life, independently of having cancer, other somatic diseases and/or mental and behavioural disorders. Suicide research should no further ignore the global consequences of intense care in older people.

## Data Availability

The datasets analysed during the current study are available from the corresponding author on reasonable request. In addition, all data are publicly available on the Switchdrive website: https://drive.switch.ch/remote.php/webdav/Documents/Suicide%20personnes%20%C3%A2g%C3%A9es.dta
